# Premartensitic transition and relevant magnetic effects in Ni_50_Mn_34_In_15.5_Al_0.5_ alloy

**DOI:** 10.1038/srep26068

**Published:** 2016-05-16

**Authors:** Yuqin Wu, Shaopu Guo, Shuyun Yu, Hui Cheng, Ruilong Wang, Haibo Xiao, Lingfang Xu, Rui Xiong, Yong Liu, Zhengcai Xia, Changping Yang

**Affiliations:** 1 Hubei Collaborative Innovation Center for Advanced Organic Chemical Materials, Hubei Key Laboratory of Ferro & Piezoelectric Materials and Devices, Faculty of Physics and Electronic Science, Hubei University, Wuhan 430062, People’s Republic of China; 2 School of Physics, Shandong University, Jinan 250100, People’s Republic of China; 3 Wuhan National High Magnetic Field Center & School of Physics, Huazhong University of Science and Technology, Wuhan 430074, People’s Republic of China; 4 School of Physics and Technology, Wuhan University, Wuhan, 430072, People’s Republic of China

## Abstract

Resistance measurement, *in situ* optical microscopic observation, thermal and magnetic measurements have been carried out on Ni_50_Mn_34_In_15.5_Al_0.5_ alloy. The existence of a pronounced premartensitic transition prior to martensitic transition can be characterized by microstructure evolution as well as exothermic peak and smooth decrease of resistance and magnetization with obvious hysteresis over a wide temperature range upon cooling. Consequently, the alloy undergoes two successive magneto-structural transitions consisting of premartensitic and martensitic transitions. Magnetoelastic coupling between magnetic and structural degrees of freedom would be responsible for the appearance of premartensitic transition, as evinced by the distinct shift of transitions temperatures to lower temperature with external applied field of 50 kOe. The inverse premartensitic transition induced by magnetic field results in large magnetoresistance, and contributes to the enhanced inverse magnetocaloric effect through enlarging the peak value and temperature interval of magnetic entropy change Δ*S*_*m*_.

Premartensitic state, referring to the intermediate state existing between the symmetric high temperature austenitic phase and a low-symmetry martensitic structure at low temperature, can be observed in the Ni-Mn-Ga ferromagnetic shape memory alloys^1^,^2^,^3^,^4^,^5^,^6^,^7^,^8^,^9^,^10^,^11^,^12^,^13^,^14^,^15^,^16^,^17^,^18^,^19^. The premartensitic phase shows approximately cubic symmetry with a micromodulated domain structure of parent phase, indicating the weak first-order nature of the premartensitic transition^2^,^3^,^4^,^5^. Anomaly of the elastic^2^,^6^,^7^,^8^,^9^, thermal^2^,^9^,^10^,^11^,^12^, resistivity^8^,^10^,^11^,^13^,^14^,^15^,^16^,^17^, and magnetic properties^3^,^7^,^8^,^9^,^10^,^11^,^12^,^15^,^16^,^17^,^18^,^19^,^20^ can be observed across this phase transition in Ni-Mn-Ga alloys. From the view of developing the application of Ni-Mn-Ga alloys, much effort has been devoted to research the origin of the premartensitic transition from parent phase to the intermediate state by theoretical and experimental methods. It is generally accepted that the premartensitic transition is a consequence of magnetoelastic coupling between the magnetic and structural degrees of freedom, as suggested by the strong magnetic field dependence of premartensitic transition temperature^3^,^8^,^9^,^11^,^13^,^16^. However, large magnetic effects associated with the premartensitic transition in Ni-Mn-Ga alloys have seldom been reported up to now due to the quite narrow temperature range and small magnetization change across the premartensitic transition. It would be attractive to obtain such behavior in material with strong magneto-structural coupling.

As a new type of ferromagnetic shape memory alloys (FSMAs), off-stoichiometric Ni_50_Mn_25+x_Z_25−x_ (Z = In, Sn, Sb) Heusler alloys have received much attention since firstly reported by Sutou *et al*.^21^. Such alloys exhibit strong magnetoelastic coupling with large magnetization difference between the ferromagnetic high-temperature austenitic phase and weak magnetic low-temperature martensitic phase. As a result, the transition temperatures of these alloys decease considerably when applying high magnetic field. Consequently, a magnetic-field-induced inverse martensitic transition (MT) from martensite to austenite occurs when a magnetic field is applied at a constant temperature close to the austenitic transition start temperature (*A*_s_). Associated with the field-induced inverse MT, a variety of interesting properties, such as magnetic shape memory effect^22^, magnetocaloric effect^23^,^24^,^25^,^26^,^27^, and magnetoresistance^28^,^29^ have been observed in the vicinity of the MT. However, there are few reports about the premartensitic transition in such Ga-free FSMAs. Recently, Ma *et al*. reported an intermediate phase transition prior to the martensitic transition and related magnetic properties, including magnetocaloric effect and magnetoresistance, in the high-pressure annealing Ni_43_Mn_41_Co_5_Sn_11_ alloy^30^,^31^. The intermediate phase transition can be evidenced by anomaly of magnetization and resistance upon heating, and the appearance of intermediate phase can be attributed to the enhancement of magnetoelastic coupling as the application of external pressure in fabrication process. Actually, chemical pressure can also be generated by relatively smaller atom substitution conveniently. Substituting the atoms in Ni–Mn–Z alloys by some smaller atom would be an effective method to get more pronounced intermediate phase or premartensitic transition behavior^25^,^26^. Since Al has the same valence electrons and smaller ionic radius compared to In, Ni_50_Mn_34_In_15.5_Al_0.5_ alloy formed by substituting In with Al was prepared. In this letter, two successive magneto-structural transitions with pronounced first-order nature, including MT and premartensitic transition, and related magnetic effects were reported.

## Results

**Figure 1:**
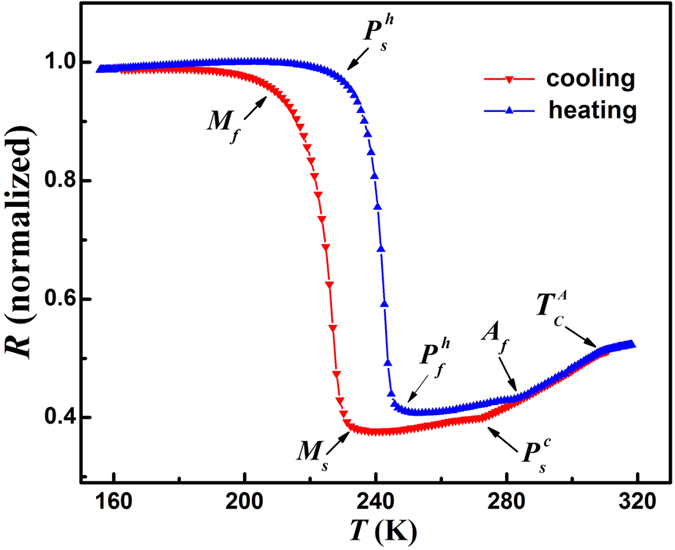
Temperature dependence of resistance *R*(*T*) for Ni_50_Mn_34_In_15.5_Al_0.5_ alloy at zero field upon heating and cooling.

Figure 1 shows the temperature dependence of resistance *R*(T) curves for Ni_50_Mn_34_In_15.5_Al_0.5_ alloy. One can note that the resistance increases sharply at about 234 K upon cooling and decreases drastically at about 250 K upon heating, indicating the MT and reverse MT. Due to the first-order nature of the MT, a thermal hysteresis about 18 K appears. Compared with the parent Ni_50_Mn_34_In_16_ alloy^28^, the Ni_50_Mn_34_In_15.5_Al_0.5_ alloy exhibits MT at higher temperature due to the chemical pressure introduced by Al doping, as reported before^26^,^27^. A change in the slope of the resistivity at 310 K without hysteresis can be recognized as the second-order magnetic transition at Curie temperature of austenite 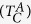. Another slope change at about 272 K, prior to MT, upon cooling with an obvious hysteresis (~14 K) can be recognized as premartensitic transition, just as reported in Ni-Mn-Ga alloys^8^,^10^,^11^,^13^,^14^,^15^,^16^. However, the premartensitic transition here appears somewhat different with those of Ni-Mn-Ga alloys, where the hysteresis are very weak and could be hardly detected. The characteristic temperatures of the reversible MT and premartensitic transition, referring to the temperatures of martensite start and finish, premartensite start upon cooling and heating, and austenite start, were marked as *M*_*s*_, *M*_*f*_, 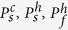 and *A*_*s*_ in the figure respectively.

**Figure 2:**
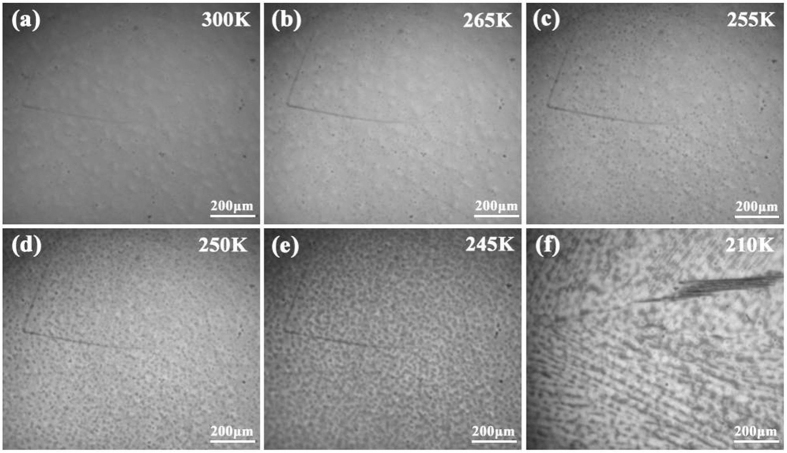
Optical micrographs at different temperatures for Ni_50_Mn_34_In_15.5_Al_0.5_ alloy upon cooling.

Since the *in situ* optical microscopic observation has been proved as a powerful method to study the microstructure evolution of samples with first-order phase transition^32^,^33^, it was employed to further confirm the existence of the premartensite phase in Ni_50_Mn_34_In_15.5_Al_0.5_ alloy. The optical micrographs at representative temperatures upon cooling are shown in Fig. 2. The sample is in austenitic phase at 300 K, exhibiting smooth surface after polish. The relative rough surface in Fig. 2(a) is resulted from being etched by dilute nitric acid, which would be good for the observation of the premartensite phase. Between 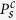 and *M*_*s*_, many tiny domains-like structures appear, and the domain density increases with decreasing temperature, as shown in Fig. 2(b–e). Similar results have been observed in Ti_50_Ni_48_Fe_2_ alloy through *in situ* dark-field image observations using electron microscopy, where the domains have been identified as premartensitic domains as a result of structural modulation of parent phase^34^,^35^,^36^. Striped fold and scaly wrinkles appear after *M*_*s*_ and keep increasing at expense of the premartensitic domains, which can be recognized as first-order MT from premartensite to martensite. Typical martensitic image at 210 K is shown in Fig. 2(f). Therefore, the phase transition prior to MT can be verified as premartensitic transition.

**Figure 3:**
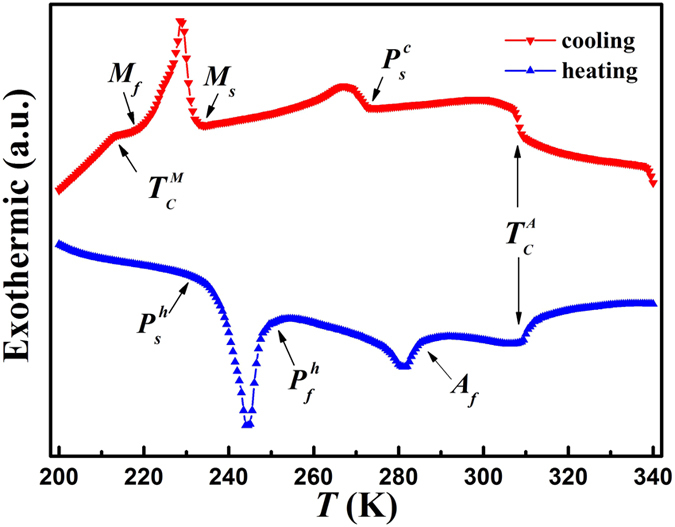
DSC curves for Ni_50_Mn_34_In_15.5_Al_0.5_ alloy upon cooling and heating.

Figure 3 shows the differential scanning calorimetry (DSC) curves for Ni_50_Mn_34_In_15.5_Al_0.5_ alloy upon cooling and heating at rete of 10 K/min. Large exothermic and endothermic peaks, corresponding to MT, premartensitic transition and their reverse transitions can be observed respectively. Thermal hysteresis clearly indicate the first-order nature of the two structural transitions. The Curie temperature of the martensite and austenite (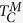 and 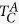) can be identified as a distinct shoulder-like feature in the DSC curves, though the small endothermic peak is too weak to be observed upon heating around 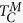. *M*_*f*_ obtained from of the *R*(*T*) curves is a little higher than that obtained from DSC curves due to the influence of 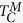. Except that, all the characteristic transition temperatures are relatively consistent.

**Figure 4:**
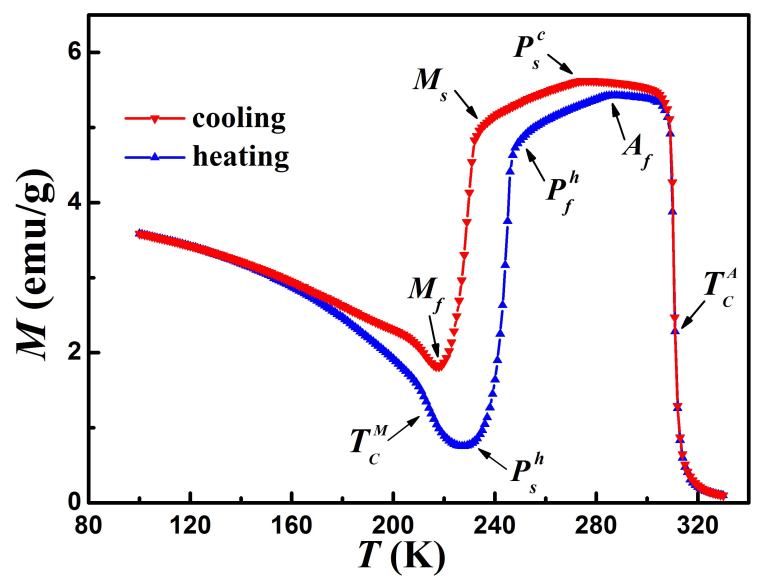
Temperature dependence of magnetization *M* (*T*) for the Ni_50_Mn_34_In_15.5_Al_0.5_ alloy measured upon cooling and heating with magnetic field of 100 Oe.

Figure 4 shows temperature dependence of magnetization *M*(*T*) curves measured upon cooling and heating processes with magnetic field of 100 Oe. All characteristic transition temperatures obtained from *M*(*T*) curves are consistent with those obtained from DSC curves. Apart from the two ferromagnetic phase transition around 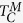 and 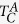, the MT and premartensitic transition exhibit two successive magnetization jump from high magnetization state to weak magnetization state upon cooling. Obvious thermal hysteresis for both transitions confirms that each transition is first-order transition and they comprise two successive magneto-structural transitions. In Ni-Mn-Ga alloys, *M*(*T*) curves exhibit two relative minimum-like anomaly kinks in quite narrow temperature interval with hardly detectable hysteresis far above MT, corresponding to premartensitic transition temperature *T*_*p*_. However, in the present case, dc magnetization and ac susceptibility decrease 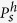 increase smoothly upon cooling/heating. Hence, it is clear that the premartensite appears around 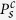 in coexistence with the austenitic phase upon cooling. With further decrease of temperature, the austenitic phase fraction gradually decreases and approaches zero around *M*_*s*_. Then the premartensite is transformed into martensite gradually from *M*_*s*_ to *M*_*f*_. Similarly, on heating, the premartensite phase appears at, and the complete transition to premartensite phase occurs at 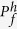. Further increasing temperature result in the transition from premartensite to austenite until full austenite phase at *A*_*f*_. In another word, the austenite and premartensite phases coexist between 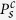 and *M*_*s*_ upon cooling as well as between 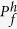 and *A*_*f*_ upon heating. The magnitude of the magnetization difference across the premartensitic transition is about 30% of that across MT (while 12% for the inverse transition), while it is only about 2% in Ni-Mn-Ga, which indicates the enhanced magnetoelastic coupling in Ni_50_Mn_34_In_15.5_Al_0.5_ alloy at high temperature prior to MT. Reasonably, the chemical pressure induced by Al doping would be responsible for the appearance of the intermediate phase.

**Figure 5:**
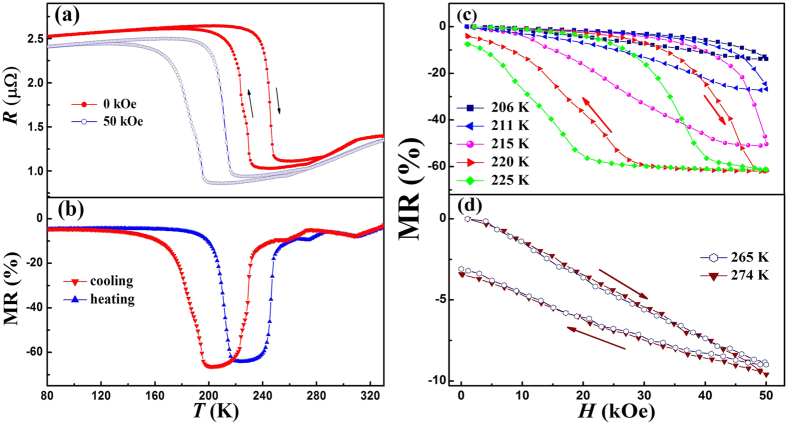
(**a**) Temperature dependence of resistance at 0 and 50 kOe for Ni_50_Mn_34_In_15.5x_Al_0.5_ alloy. (**b**) MR under a magnetic field of 50 kOe upon heating and cooling. (**c,d**) Field dependence of the MR at selected temperatures. Arrows indicate the direction of temperature and field variation.

With the large magnetization difference realized in the two successive magneto-structural transitions, we thus expect it to manifest a field-induced inverse transition. Figure 5(a) shows the temperature dependence of resistance *R*(*T*) curves for Ni_50_Mn_34_In_15.5_Al_0.5_ alloy measured under zero field and 50 kOe. The *R*(T) curves under 50 kOe are found to be similar to that under zero field, but the phase transition temperatures notably shift to lower temperature under 50 kOe. The *M*_s_ is decreased by 34 K while 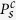 is decreased by 15 K. The large field dependences of transition temperatures can be attributed to the field-induced reverse MT, which further confirms that the two successive phase transitions originate from magnetoelastic coupling. Thus, large MR effects can be expected around the phase transitions. The MR was calculated as (*R*_*H*_*-R*_*0*_)*/R*_*0*_, where *R*_*H*_ is the resistivity under magnetic field and *R*_0_ is the resistivity at zero field. Figure 5(b) displays the temperature dependence of MR under 50 kOe. Upon heating, three negative peaks at 225 K, 274 K and 310 K with the value of 64%, 9.5%, and 8%, respectively, can be observed in the regions of MT, premartensitic transition and ferromagnetic-paramagnetic transition. The difference of MR upon cooling and heating in the regions of MT at low temperature is likely due to the different fractions of coexistent premartensite and martensite, as reported in conventional MT in the Ni-Mn based alloys. Similarly, the difference of MR upon cooling and heating in the regions of premartensitic transition at low temperature can be attributed to the different fractions of coexistent premartensite and austenite during premartensitic transition and the reverse one. In order to get a better understanding of the MR effects, isothermal MR curves are shown in increasing and decreasing magnetic fields at different temperatures, as described in inset of Fig. 5(c,d). For the measurements, firstly the samples were cooled down to 160 K from 340 K and then the temperature went up to the desirable temperature to perform magnetic field dependence of resistance. Between 205 K and 225 K, the alloy is in the martensitic structure. Increasing magnetic field should induce the inverse MT from martensite to premartensite, and hence a sharp change in MR is observed in Fig. 5(c). The MR at low temperatures (205 K, 210 K and 215 K) can return to its original value of 0% after a field cycle up to 50 kOe, exhibiting a fully reversible behavior. However, the MR does not recover completely after a complete magnetization cycle at 220 K and 225 K. The magnitude of the irreversible MR increases with increasing temperature, indicating that the amount of remnant premartensite increase with temperature. This demonstrates that the magnetic field-induced austenite cannot be restored to martensite completely after demagnetization. At 265 K and 275 K, we get coexisting premartensite and austenitic phases in the sample. The magnetic-field-induced reverse premartensitic transition at the two temperatures can be indicated by the large magnetic hysteresis, shown in Fig. 5(d). The slow change of MR can be ascribed to the similar structure between the premartensite and austenite. Additionally, the irreversible behavior can observed, implying that the complete return transition (austenite to premartensite) cannot be realized.

**Figure 6:**
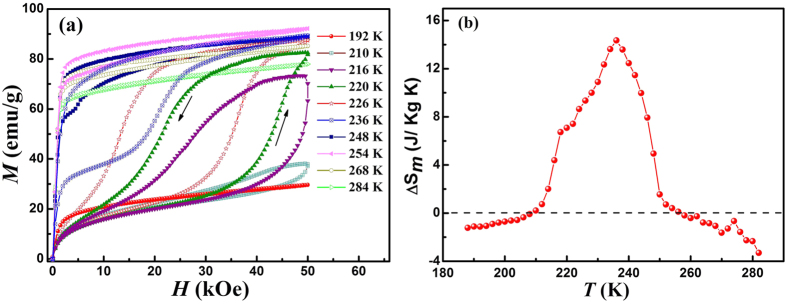
(**a**) Isothermal magnetization *M*(*H*) curves for Ni_50_Mn_34_In_15.5_Al_0.5_ alloy at selected temperatures. (**b**) Temperature dependence of isothermal magnetic entropy change Δ*S*_*m*_ in the magnetic field of 50 kOe. Arrows indicate the direction of field variation.

The isothermal magnetization *M*(*H*) curves at different temperatures with the field up to 50 kOe were measured, and the temperature step is 2 K. Figure 6(a) shows the typical *M-H* curves for the sample. Before each measurement, the sample was first cooled to 150 K (well below *M*_*f*_) to ensure the same initial state of a fully transformed martensite and then heated to the measurement temperature. No visible hysteresis can be observed in the *M*(*H*) curves at temperatures above 284 K (in the austenitic phase) and below 190 K (in the martensitic phase), which can be attributed to the absence of field-induced phase transition. The filed-induced transition from martensite to premartensite can be indicated by metamagnetic behavior and magnetic hysteresis between field-up and field-down processes. From Fig. 6(a), we can find that the premartensite start temperature under 50 kOe upon heating is at 220 K, which means application of 50 kOe can induce the complete transition from martensite to premartensite. Hence, with the increasing of austenite fraction at higher temperatures between 220 K and *A*_*s*_ (250 K), application of 50 kOe can not only induce transition from martensite to premartensite, but also induce transition from premartensite to austenite, which can be confirmed by the increasing of magnetization at 50 kOe from 220 K to 248 K, though there are no second metamagnetic behaviors. In accordance with the result of Fig. 5(d), the transition from premartensite to austenite can be displayed by the magnetic hysteresis in the representative curves at 254 K and 268 K.

The isothermal magnetic entropy change Δ*S*_*m*_ as a function of temperature and magnetic field can be calculated from magnetization isotherms using Maxwell relation 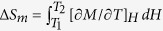. Figure 6(b) shows the temperature dependence of Δ*S*_*m*_ in the magnetic field of 50 kOe. The spike-like behavior indicates the peak value (14.4 J/kg K) may be overestimated due to the application of Maxwell relation for first-order magnetostructural transition^37^,^38^. Nevertheless, such result can still be employed to study the origin of Δ*S*_*m*_. In previous system with two successive magneto-structural transitions, either two-step MT^39^ or MT and preceding intermediate phase transition^30^, two positive Δ*S*_*m*_ peaks can be observed, corresponding to the two field-induced inverse phase transitions, respectively. In the present case, only one Δ*S*_*m*_ peak can be observed. According to the above analysis, the two-step inverse phase transition from martensite to premartensite, and then to austenite can be induced successively when applying magnetic field of 50 kOe at a constant temperatures between 220 K and 250 K. So both the two inverse phase transition induced by magnetic field contribute to the large value and wide temperature span of the peak, thus enhancing the magnetocaloric effect effectively.

## Discussion

Premartensitic transition has been intensively investigated in Ni-Mn-Ga alloys for decades. However, owing to the small magnetization change and narrow temperature range across the premartensitic transition, the magnetic effects have seldom been reported. Since the occurrence of premartensitic transition in Ni-Mn-Ga alloys is a consequence of magnetoelastic coupling. The Ga-free Ni–Mn based FSMAs exhibit strong magnetoelastic coupling across MT, making them as excellent candidates to study the interesting properties associated with premartensitic transition. Intermediate phase transition behaviors were observed in high-pressure annealing Ni–Mn–Co–Sn alloys, which can be attributed to the enhancement of magnetoelastic coupling at high temperatures prior to MT, while the enhancement of magnetoelastic coupling is resulted from the chemical pressure generated in the synthetizing process. Such chemical pressure can also be induced by smaller atom substitution. Here, in Ni_50_Mn_34_In_15.5_Al_0.5_ alloy formed by substituting In with smaller Al, pronounced premartensitic transition was detected by resistivity, *in situ* microscopic observation calorimetric and magnetic measurements, and the nature of first-order was displayed by thermal/ magnetic hysteresis. Our results indicate that smaller atom substitution would be an effective method to get more pronounced premartensitic transition behavior in Ni–Mn based FSMAs.

In summary, two successive magneto-structural transitions consisting of martensitic and premartensitic transitions were observed in Ni_50_Mn_34_In_15.5_Al_0.5_ alloy. The first-order nature of premartensitic transition can be evinced by microstructure evolution and large thermal/magnetic hysteresis. Owing to magnetoelastic coupling, the transitions temperatures shift to lower temperature with the external applied field. Large magnetoresistance and enhanced magnetic entropy change Δ*S*_*m*_ can be obtained associated with the field-induced inverse phase transitions.

## Methods

The bulk Ni_50_Mn_34_In_15.5_Al_0.5_ alloy of nominal composition were prepared by arc-melting the required amount of constituent high purity (99.99% purity) elements in a cold copper crucible under argon atmosphere protection. An additional 2 wt.% Mn was added to compensate for evaporation losses. Homogenization was achieved by sealing the ingot under argon atmosphere in quartz ampoules and annealing at 1073 K for 24 h, followed by quenching in ice water. The sample used for imaging was polished and subsequently etched by dilute nitric acid. The *in situ* microscopic observation was performed using a magneto-optical imaging system (MOIS) attached with an ultrahigh speed camera. Heat flow data were collected by differential scanning calorimeter (DSC, Q2000, TA) on modulated mode with a cooling/heating rate of 10 K/min. The magnetic properties were measured by a superconducting quantum interference device (SQUID-VSM, Quantum Design) magnetometer and electrical transport properties were measured in a physical property measurement system (PPMS, Quantum Design).

## Additional Information

**How to cite this article**: Wu, Y.Q. *et al*. Premartensitic transition and relevant magnetic effects in Ni_50_Mn_34_In_15.5_Al_0.5_ alloy. *Sci. Rep.* **6**, 26068; doi: 10.1038/srep26068 (2016).
